# Supplementary figures and images for: A Systematic Review of Fumagillin Field Trials for the Treatment of Nosema Disease in Honeybee Colonies
Source: Insects. 2024 Jan 2;15(1):29. doi: 10.3390/insects15010029 (PMC10816105; doi:10.3390/insects15010029)

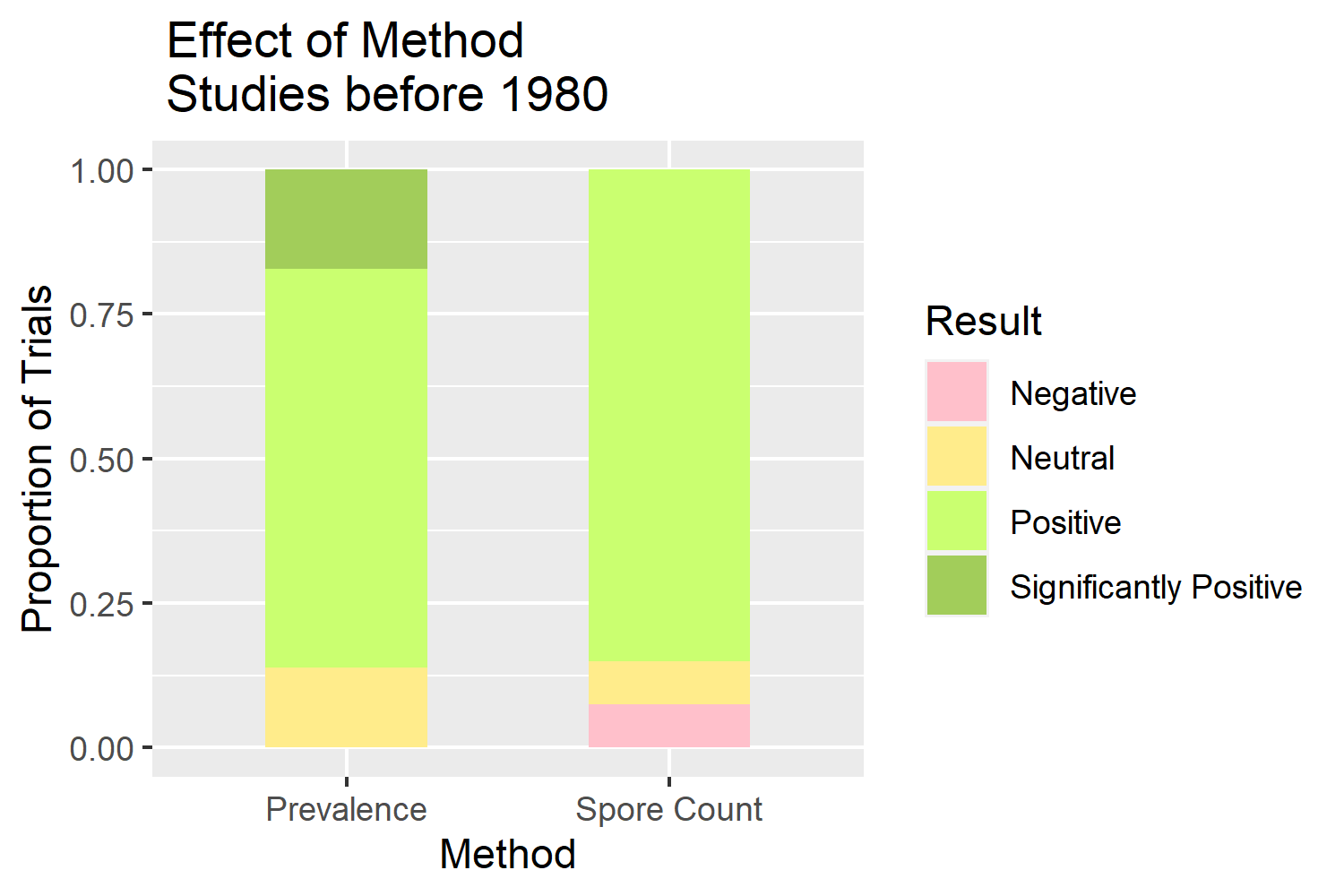

Supplement: Supplementary file 1 [file insects-15-00029-s001.zip › Figure S1.tiff]

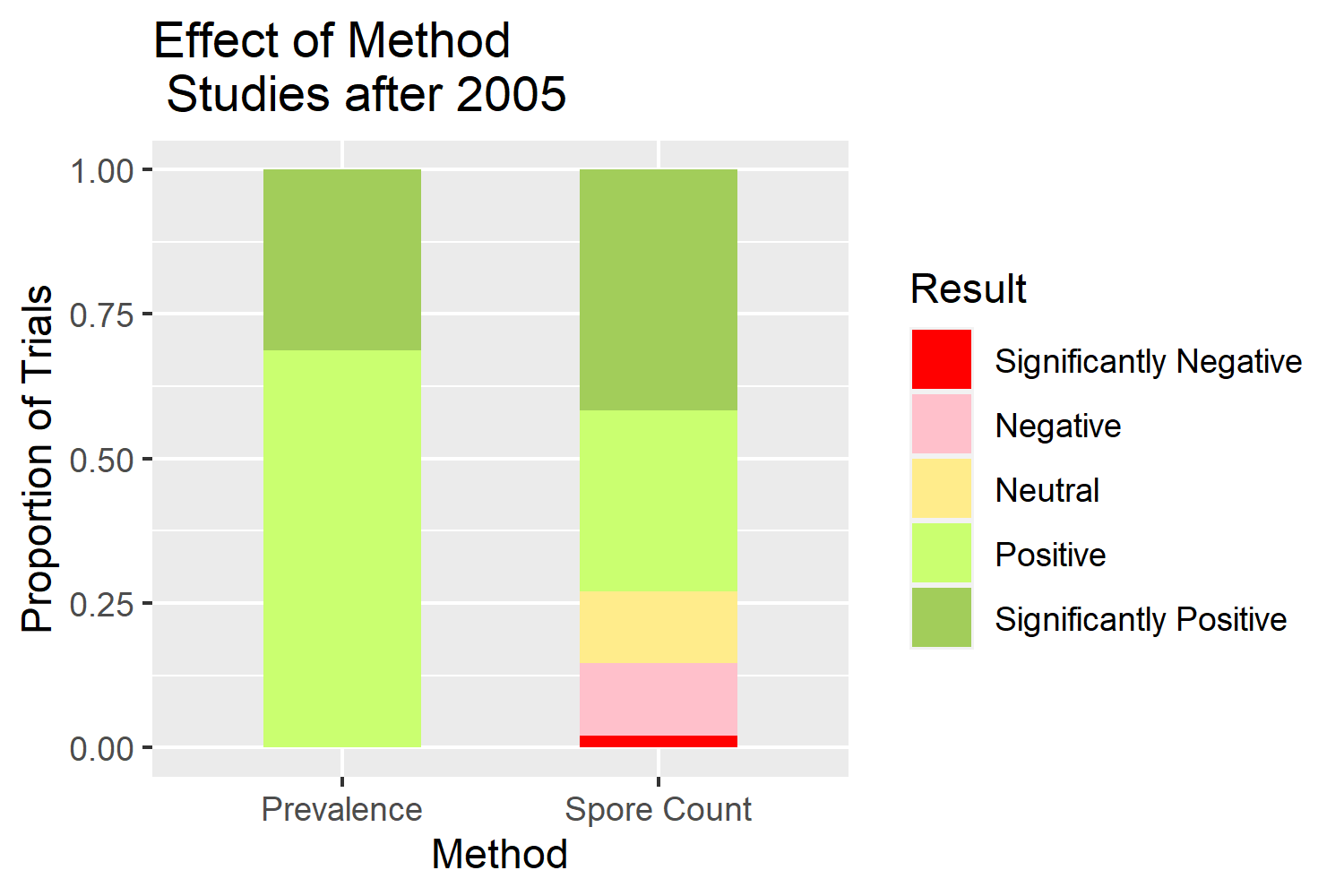

Supplement: Supplementary file 1 [file insects-15-00029-s001.zip › Figure S2.tiff]

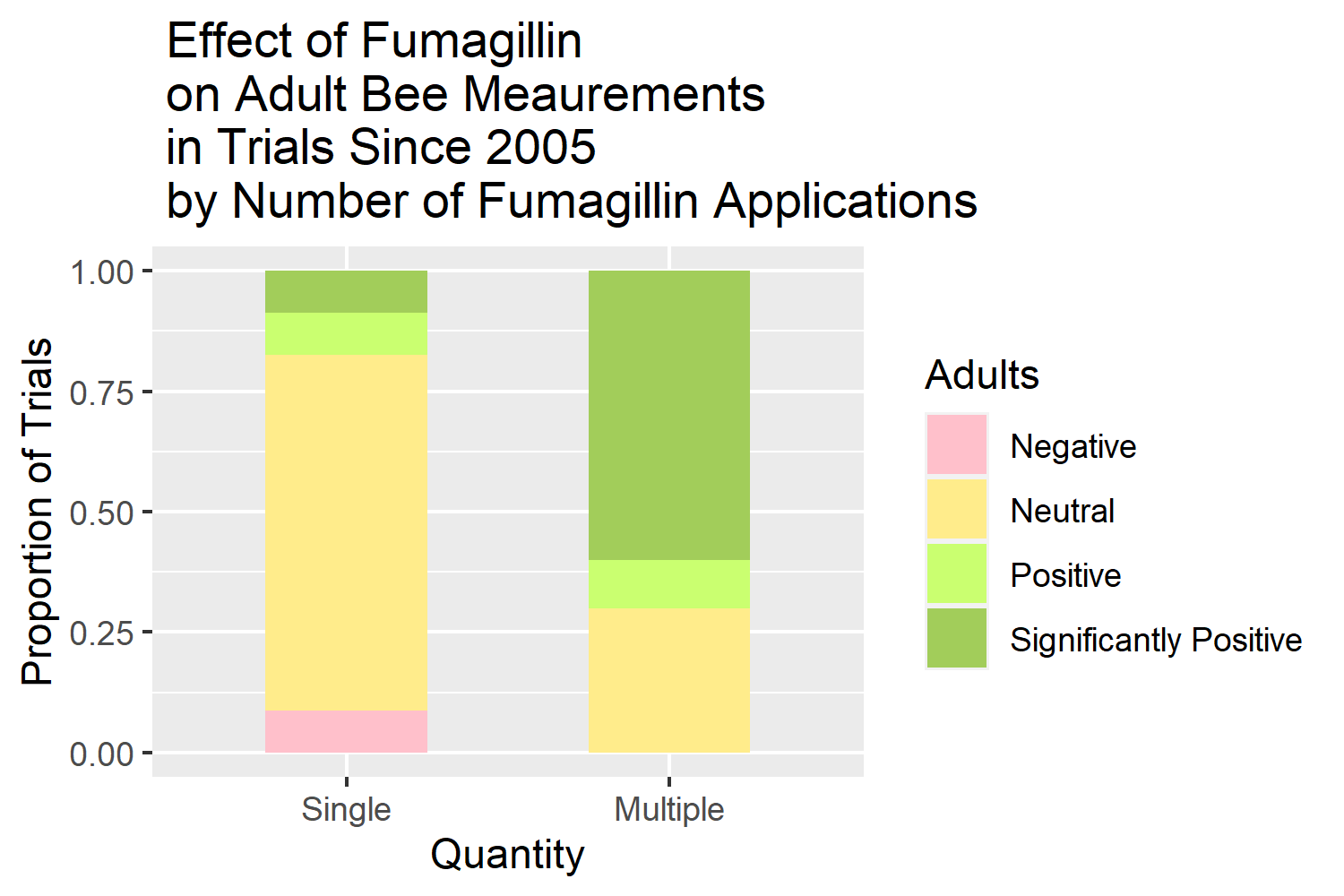

Supplement: Supplementary file 1 [file insects-15-00029-s001.zip › Figure S3.tiff]

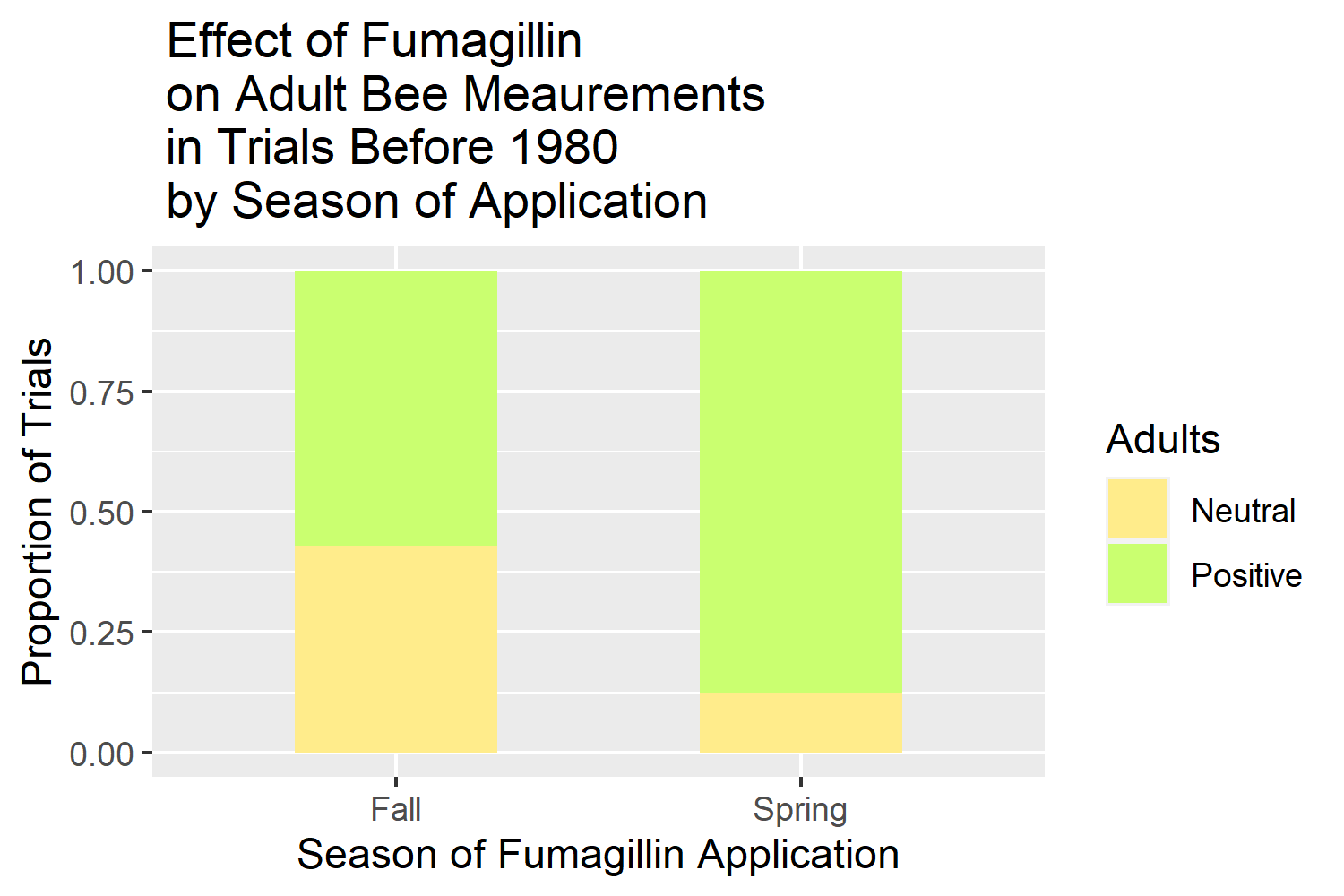

Supplement: Supplementary file 1 [file insects-15-00029-s001.zip › Figure S4.tiff]
